# Discovery and Evolution of a Divergent Coronavirus in the Plateau Pika From China That Extends the Host Range of Alphacoronaviruses

**DOI:** 10.3389/fmicb.2021.755599

**Published:** 2021-10-07

**Authors:** Wentao Zhu, Jing Yang, Shan Lu, Dong Jin, Shusheng Wu, Ji Pu, Xue-lian Luo, Liyun Liu, Zhenjun Li, Jianguo Xu

**Affiliations:** ^1^State Key Laboratory of Infectious Disease Prevention and Control, National Institute for Communicable Disease Control and Prevention, Chinese Center for Disease Control and Prevention, Beijing, China; ^2^Shanghai Public Health Clinical Center, Fudan University, Shanghai, China; ^3^Research Units of Discovery of Unknown Bacteria and Function, Chinese Academy of Medical Sciences, Beijing, China; ^4^Yushu Prefecture Center for Disease Control and Prevention, Yushu, China; ^5^Research Institute of Public Heath, Nankai University, Tianjin, China

**Keywords:** plateau pika (ochotona curzoniae), coronavirus, *alphacoronavirus*, ancestry, evolution

## Abstract

Although plateau pikas are the keystone species in the plateau ecosystem of the Qinghai Province of China, little is known about their role in the evolution and transmission of viral pathogens, especially coronaviruses. Here, we describe the characterization and evolution of a novel alphacoronavirus, termed plateau pika coronavirus (PPCoV) P83, which has a prevalence of 4.5% in plateau pika fecal samples. In addition to classical gene order, the complete viral genome contains a unique nonstructural protein (NS2), several variable transcription regulatory sequences and a highly divergent spike protein. Phylogenetic analysis indicates that the newly discovered PPCoV falls into the genus *Alphacoronavirus* and is most closely related to rodent alphacoronaviruses. The co-speciation analysis shows that the phylogenetic trees of the alphacoronaviruses and their hosts are not always matched, suggesting inter-species transmission is common in alphacoronaviruses. And, PPCoV origin was estimated by molecular clock based on membrane and RNA-dependent RNA polymerase encoding genes, respectively, which revealed an apparent discrepancy with that of co-speciation analysis. PPCoV was detected mainly in intestinal samples, indicating a potential enteric tropism for the virus. Overall, this study extends the host range of alphacoronaviruses to a new order (Lagomorpha), indicating that plateau pikas may be the natural reservoir of PPCoV and play an important and long-term role in alphacoronavirus evolution.

## Introduction

Over past decades, coronaviruses have caused circulating epidemics and worldwide pandemics, including the most recent pandemic caused by the novel coronavirus (SARS-CoV-2; Jiang and Shi, 2020; Liu et al., 2021). Coronaviruses are spherical, single-stranded, positive-sense, enveloped RNA viruses with genome sizes ranging from 27 to 32 kilobases (kb) in length (Schoeman and Fielding, 2019; Haake et al., 2020). They are classified into four genera: *Alphacoronavirus*, *Betacoronavirus*, *Gammacoronavirus*, and *Deltacoronavirus*, belonging to the family *Coronaviridae* (Wang et al., 2019; Haake et al., 2020; Zhu et al., 2021a). Alphacoronaviruses originate from bats and predominantly infect mammals, including shrews (*Sorex araneus* and *Suncus murinus*), ferrets, pigs, cats, and a wide variety of rats (Woo et al., 2012; Wang et al., 2017; Wu et al., 2018; Haake et al., 2020). *Alphacoronavirus* contain 19 species in 14 subgenera and are capable of causing mild to lethal diseases in humans and animals. Two human coronaviruses (HCoV NL63 and HCoV 229E) cause mild illnesses; however, four porcine coronaviruses [swine acute diarrhea syndrome coronavirus (SADS-CoV), transmissible gastroenteritis virus (TGEV), porcine epidemic diarrhea virus (PEDV), and porcine respiratory coronavirus (PRCV)] cause watery diarrhea, gastroenteritis, severe villous atrophy, and high mortality in piglets (van der Hoek et al., 2006; Yip et al., 2016; Zhou et al., 2018; Wang et al., 2019; Cheng et al., 2020; Haake et al., 2020; Jung et al., 2020).

Coronaviruses have the largest genomes (26 to 32kb) among RNA viruses, with gene order of 5'-leader-UTR to 3'UTR-poly (A) tail is replicase (ORF1ab)-spike (S)-envelope (E)-membrane (M)-nucleocapsid (N; Wang et al., 2017, 2020). Coronavirus genomes encode three classes of proteins, including 16 nonstructural proteins (nsp1–nsp16) from ORF1ab, four major structural proteins (S, E, M, and N) and several accessory proteins between these structural proteins (Haake et al., 2020). Recently, a predicted NS2 between ORF1ab and S was discovered in rodent alphacoronaviruses within subgenus *Luchacovirus*. NS2 is not present in any other characterized alphacoronaviruses, but is usually found in betacoronaviruses (Lau et al., 2012b; Tsoleridis et al., 2019).

The plateau pika (*Ochotona curzoniae*), also known as the black-lipped pika, belongs to family Ochotonidae within order Lagomorpha and is a keystone species of the Tibetan Plateau of China (Yang et al., 2011; Wu et al., 2019). They are small, non-hibernating, rabbit-like mammals with short limbs, rounded ears, no external tail, and inhabiting areas around 3,100 to 5,000 meters above sea level throughout the Tibetan Plateau of China (Yang et al., 2011; Yu et al., 2014; Su et al., 2016). Recently, plateau pikas have been considered as hosts for influenza viruses (H7N2, H9N2 and H5N1; Zhou et al., 2009; Yu et al., 2014; Su et al., 2016). Additional viruses from plateau pikas are rarely reported. Although betacoronaviruses and deltacoronaviruses have been identified in other wild animals in the Qinghai-Tibetan Plateau (Wu et al., 2018; Zhu et al., 2021b), little is known about the role of plateau pikas in the evolution and transmission of coronaviruses. In this study, we identified a novel coronavirus in the plateau pika from the Tibetan Plateau of China and explored the evolution and virus-host co-divergence within the genus *Alphacoronavirus*.

## Materials and Methods

### Samples

In July 2019, a total of 157 plateau pikas were captured from four sites (3,890–3,970 meters above sea level) in mountainous regions of Yushu Tibetan Autonomous Prefecture in Qinghai Province of China. The distance between sample collection sites is about 50 kilometers. All captured animals were clinically healthy and identified through morphological examination. Pikas were trapped and euthanized, and their respiratory tracts and intestinal contents were removed and preserved in 5-ml tubes directly. All sampling work was approved by the ethics committee of the National Institute for Communicable Disease Control and Prevention of China CDC (ICDC-2019012), and conducted by Yushu Prefecture Center for Disease Control and Prevention as part of plague surveillance.

### RNA Extraction and Coronavirus Screening

Viral RNA of each fecal sample was extracted using QIAamp Viral RNA Mini kit (QIAGEN) and resuspended in 50μl of DNase-free, RNase-free water. Conserved primers were used to amplify the 440-bp fragment of RNA-dependent RNA polymerase (RdRp) of coronaviruses (Woo et al., 2005). Reverse transcription-polymerase chain reaction (RT-PCR) was performed by PrimeScript™ One Step RT-PCR Kit Ver.2 (Takara) with 50°C for 30min and 33cycles of 94°C for 30s, 48°C for 30s, 72°C for 30s, and a final extension at 72°C for 10min. The PCR products were gel-purified before conducting Sanger sequencing.

### Complete Genome Sequencing

The total RNA of a coronavirus positive sample was firstly rRNA-removed using the Ribo-Zero Gold rRNA Removal Kit. The TruSeq stranded total RNA library prep gold kit was used for library construction according to instructions. Paired reads (2×150bp) were obtained using the Illumina HiSeq2000 platform, which were assembled into contigs using Trinity v2.8 (Grabherr et al., 2011). The assembled contigs were annotated in NCBI non-redundant protein database using Diamond (Buchfink et al., 2015). To confirm the assembled genome, clean data were mapped back to the genome using Bowtie 2 (Langmead and Salzberg, 2012) and assembled using SPAdes (Bankevich et al., 2012). Moreover, several primers were designed to amplify the NS2, *S*, and *N* genes followed by Sanger sequencing (Supplementary Table S2).

### Genome and Phylogenetic Analyses

The deduced amino acid and nucleotide sequences of the open reading frames (ORFs) were found using ORF finder with default parameters and compared to those of other coronaviruses (Gao et al., 2003). The proteinase cleavage sites of CoVs were predicted using an online tool (Gao et al., 2003). The amino acid and nucleotide identities were calculated using BioAider version 1.334 (Zhou et al., 2020). Protein families and homology searches were performed by InterPro (Blum et al., 2021) and HMMER (Potter et al., 2018), respectively. Transmembrane helices in proteins were predicted by TMHMM with a hidden Markov model (Krogh et al., 2001). N-linked glycosylation and O-GalNAc (mucin type) glycosylation sites were predicated using web server.^1^ The amino acid sequences were aligned using the MAFFT program (Katoh and Standley, 2013). Aligned regions were polished using Gblocks (Talavera and Castresana, 2007). Phylogenetic analyses were constructed using the maximum-likelihood method by PhyML 3.0 with IBV as the outgroup and bootstrap values of 1,000 (Guindon et al., 2010). Based on AIC criteria, the substitution models were selected using ModelFinder (Kalyaanamoorthy et al., 2017).

### Estimation of Divergence Dates

The complete RdRp and M genes of PPCoV and related coronaviruses were acquired and aligned using the MAFFT program (Katoh and Standley, 2013). Sampling times were collected, and the divergence was estimated using a Bayesian Markov chain Monte Carlo (MCMC) method implemented in BEAST v 1.10 (Lau et al., 2012a). Analyses were performed using the SRD06 substitution model with uncorrelated relaxed clock. The MCMC run was 1×10^8^ steps long, with sampling every 1,000 steps. The mean time of the most recent common ancestor (tMRCA) and the highest posterior density regions at 95% (HPDs) were calculated. All the ESS values of statistic parameters were greater than 200 and visualized using Tracer v1.7.1 (Suchard et al., 2001). The trees were summarized in a target tree (a 10% burn-in) by the Tree Annotator program included in the BEAST package.

### Virus-Host Co-divergence and Recombination Detection

The host species were collected and used to infer phylogenetic trees of alphacoronavirus mammal hosts using TimeTree (Kumar et al., 2017). A phylogenetic virus tree was built based on polyproteins as described above. A virus-host co-divergence tree was constructed using Jane software package (Conow et al., 2010) with generations set at 100 and population size set at 100. Event costs were set at 0 for co-divergence, 1 for duplications, 1 for host-switching, and 1 for loss. Recombination analysis was conducted using both Simplot version 3.5.1 with setting parameters of the F84 model, window size 1,000bp and step 200bp (Lau et al., 2012b) and Recombination Detection Program (RDP) version 4.97 with default parameters (Martin and Rybicki, 2000).

## Results

### Novel Alphacoronavirus in Plateau Pikas

A total of 157 plateau pikas were obtained from four sites in Qinghai-Tibet plateau (Qinghai Province) in the northwest of China. RT-PCR targeting a 440bp fragment of the RdRp gene was conducted to screen potentially novel coronaviruses (CoVs). Viral RNA was positive in seven (4.5%) of 157 intestinal samples, with only positive in two respiratory samples of these seven pikas. All seven positive intestinal samples were collected from the same site, with overall detection rate of 13.7% at this site. Sequences of the screening PCR products revealed that they had <85.8% nucleotide identities to the corresponding sequences of known alphacoronaviruses, with 100% nucleotide identity to each other. Primer for multiple genome sites including *NS2*, *S*, and *N* genes were used to confirm the genome of all the positive samples, showing 100% nucleotide similarity among them. These results indicated that a new CoV (sharing <85.8% nucleotide identities to known alphacoronaviruses based on the 440bp fragment) circulates in plateau pikas of this site. No obvious diseases were observed in plateau pikas infected with the new CoV. To further confirm the pathogenesis, sections of respiratory tracts of plateau pikas that were positive for CoV were checked by staining with hematoxylin–eosin (HE). No inflammation was observed in respiratory samples (Supplementary Figure S1).

### Complete Genome Characterization

**Figure 1:**
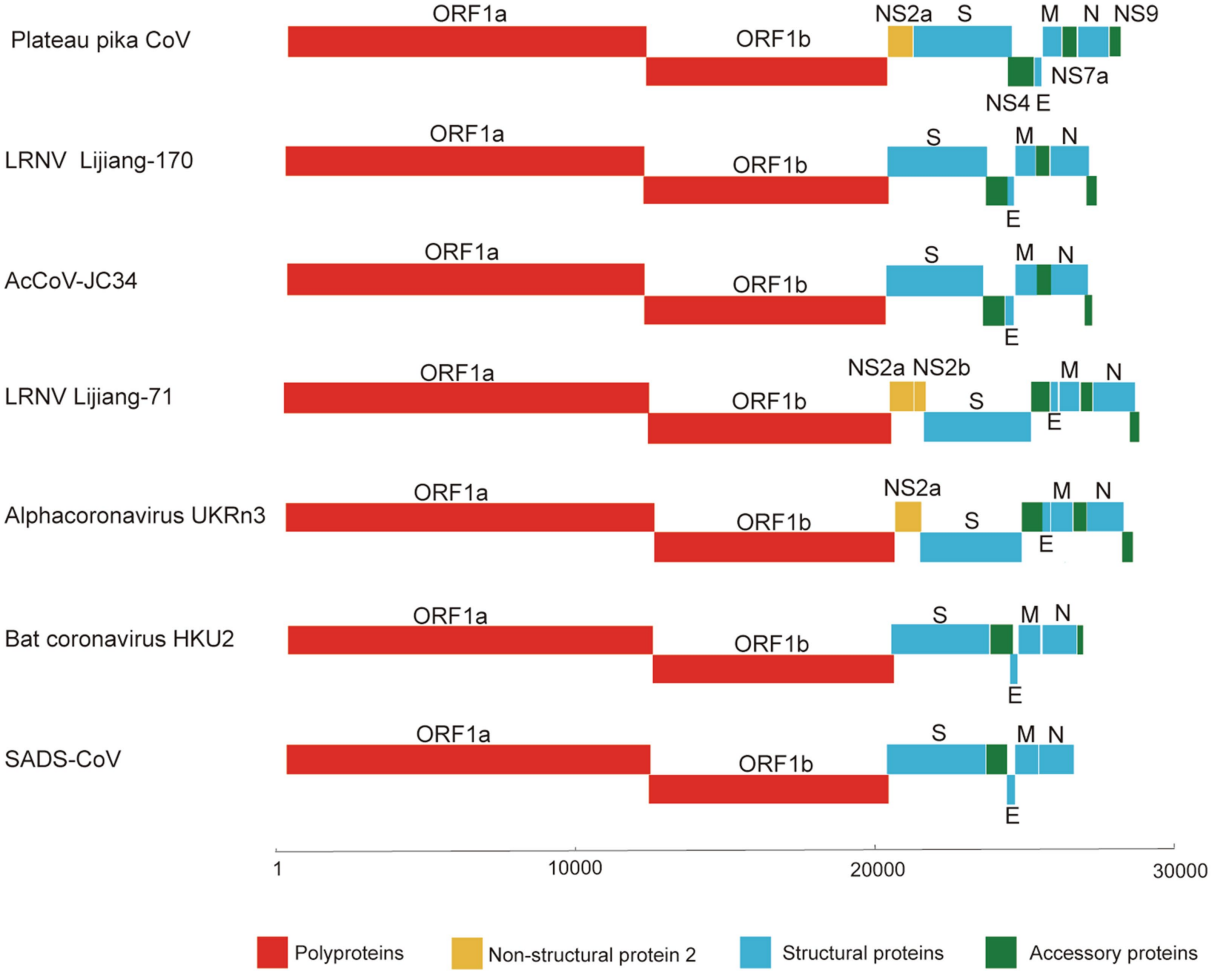
Schematic diagram of the genomes of PPCoV and related representative alphacoronaviruses. LRNV, Lucheng Rn rat coronavirus; AcCoV-JC34, Coronavirus AcCoV-JC34; SADS-CoV, Swine acute diarrhea syndrome coronavirus.

Since partial RdRp sequences indicated that the CoV may represent a new member of the genus *Alphacoronavirus*, we selected one (designated P83) of the CoV positive samples for high-throughput sequencing. A total of 144,123,796 reads were obtained. To verify the assembled viral genome, a total of 154,419 reads were mapped back to the coronavirus genome. The genome was temporarily named plateau pika coronavirus (PPCoV) P83 (GenBank accession number MZ577265) with a size of 28,312 nucleotides (without 5' and 3' rapid amplification) and 35.8% G+C content. The genome organization of PPCoV was similar to that of other related alphacoronaviruses, with classical gene order of 5'-replicase ORF1ab-spike (S)-envelope (E)-membrane (M)-nucleocapsid (N)-3' (Figure 1; Table 1). A putative transcription regulatory sequence (TRS) motif of 5'-AACUAA-3' was located upstream in majority of the genes (Table 1), which was similar to that in other alphacoronaviruses. Some variations of TRS motif were observed in the PPCoV genome for *NS2a* (5'-TACUUUAA-3'), *E* (5'-UACUAA-3'), and *NS7a* (5'-AAGUAA-3') genes (Table 1), but no TRS motif was observed for *NS9* gene. The putative nonstructural proteins (nsp) from ORF1ab with their corresponding cleavage sites are listed in Supplementary Table S1. The lengths of nsp2 and nsp4 in PPCoV differed from those of related alphacoronaviruses (Supplementary Table S1).

**Table 1:** Coding potential and putative transcription-regulatory sequences (TRS) of PPCoV.

	Location (nt)	Length (nt)	Length (aa)	Frames(s)	TRS sequence
ORF1ab	316–20,243	19,929		+1, +3	AACUAA(249)AUG
NS2a	20,246–21,079	834	277	+2	TACUUUAA(82)AUG
S	21,095–24,457	3,363	1,120	+2	AACUAA(3)AUG
NS3	24,243–25,103	861	286	+3	UACUAA(572)AUG
E	25,103–25,339	237	78	+2	UACUAA(48)AUG
M	25,349–26,095	747	248	+2	AACUAA(1)AUG
NS7a	26,131–26,610	480	159	+1	AAGUAA(108)AUG
N	26,625–27,803	1,179	392	+3	AACUAA(1)AUG
NS9	27,631–28,044	414	137	+1	

The genome of PPCoV showed 69.2 to 71.4% nucleotide sequence identities to Lucheng Rn rat coronaviruses (LRNVs), and <50.7% to other alphacoronaviruses (Table 2). The ORF1ab of PPCoV shared 75.1 to 76.2% amino acid (aa) identities with the ORF1ab of LRNVs, and <48.6% with that of other CoVs. The replicase domains of 3C-like protease (3CLpro), 3'-to-5' exonuclease (ExoN), nidoviral uridylate-specific endoribonuclease (NendoU) possessed <62.9%, <86.2%, and <85.0% aa identities, respectively, to the corresponding domains of other CoVs (Table 2).

**Table 2:** Sequence identity and genome information for PPCoV compared with related alphacoronaviruses.

				Pairwise amino acid identity (%)								
Coronavirus	Genome size	G+C content	Pairwise genome identity	3CLpro	RdRp	Hel	ExoN	NendoU	NS2a	S	M	N
Lucheng Rn rat coronavirus												
AcCoV-JC34	27,682	40.1	69.2	62.9	92.9	93.5	84.2	83.0		72.7	84.7	67.4
AlphaCoV UKRn3	28,763	40.2	70.7	61.5	93.2	93.6	85.6	83.5	53.2	69.5	85.5	70.7
LRNV Lijiang-170	27,563	40.2	69.5	62.9	91.9	93.1	84.0	83.2		73.0	84.3	68.2
LRNV Lucheng-19	28,763	40.3	70.7	61.2	93.2	93.6	86.2	84.7	53.1	70.7	86.7	70.7
RtMruf-CoV-1/JL2014	29,197	39.8	70.8	62.3	93.8	93.6	86.0	85.0	55.8	70.6	87.6	69.3
RtRl-CoV/FJ2015	28,722	40.3	71.4	61.5	93.1	93.5	85.6	84.7	53.6	69.6	86.4	71.0
Mink coronavirus	28,941	37.5	44.4	25.6	76.9	72.5	64.2	59.5		18.3	51.1	29.1
Ferret coronavirus	28,434	39.0	44.2	24.6	76.5	72.0	64.0	59.3		17.3	48.2	31.2
Feline coronavirus	28,595	38.7	43.1	25.9	74.7	70.8	63.2	58.4		17.4	50.4	28.0
TGEV	28,586	37.6	44.3	26.5	74.8	71.6	63.8	59.0		17.4	51.5	29.6
Human coronavirus 229E	27,317	38.3	45.2	23.7	73.0	73.0	67.6	58.7		19.1	45.0	24.8
Human coronavirus NL63	27,553	34.5	47.3	23.7	73.4	72.5	64.7	60.8		18.2	49.4	29.2
SADS-CoV	27,173	39.4	50.5	23.3	74.9	72.7	66.0	62.4		39.4	51.4	32.3
Wencheng Sm shrew CoV	26,028	31.5	50.7	19.8	68.0	67.2	59.0	50.0		31.3	45.2	24.8
Shrew-CoV/Tibet2014	27,102	36.6	39.1	16.9	63.8	62.6	55.8	49.4		29.0	33.2	27.1

LRNV, Lucheng Rn rat coronavirus; AcCoV-JC34, Coronavirus AcCoV-JC34; RtMruf-CoV-1/JL2014, Rodent coronavirus isolate RtMruf-CoV-1/JL2014; RtRl-CoV/FJ2015, Rodent coronavirus isolate RtRl-CoV/FJ2015; TGEV, porcine transmissible gastroenteritis virus; SADS-CoV, Swine acute diarrhea syndrome coronavirus.

*NS2a* gene between *ORF1ab* and *S* genes was minimally present in rat coronaviruses with exception of alphacoronavirus UKRn3, LRNV Lucheng-19, RtMruf-CoV-1/JL2014, and RtRl-CoV/FJ2015 (Table 2). An *NS2a* gene (20,246–21,079nt) was predicated in PPCoV with 277 aa (Table 1); it had 53.1 to 55.8% aa identities to related alphacoronaviruses (Table 2) and <41.0% aa identities to corresponding domains of betacoronaviruses. Multiple alignments of amino acid sequences of NS2a are shown in Supplementary Figure S2. Analyses indicated that NS2a of PPCoV contained no membrane helices, with N-glycosylated sites at 149 and 181, and no O-glycosylation sites. An InterProScan search showed that NS2a had homologs to the cyclic phosphodiesterase superfamily. However, the definite function of NS2a in CoVs remain unclear.

Although showing similar structures to those of related CoVs (Supplementary Figure S3), S protein of PPCoV shared only 69.5 to 73.0% aa identities with LRNVs, and <43.4% aa identities with other CoVs. A transmembrane domain (from residues 1,063 to 1,085) was predicated in the S protein, with most of the protein (residues 1 to 1,062) on the outside of the virus and a cytoplasmic tail (residues 1,086 to 1,120), which was similar to that of the Wencheng Sm shrew CoV and bat coronavirus HKU10 (Lau et al., 2012a; Wang et al., 2017). Meanwhile, N-glycosylated modifications of S protein of PPCoV were detected in 13 sites (93, 150, 190, 300, 326, 540, 657, 778, 919, 981, 997.1,010, and 1,050).

Other predicted proteins of NS3, E, M, NS7a and N proteins of PPCoV showed a difference (<90% aa identities) to corresponding proteins of other alpha CoVs, especially in NS8 (<40% aa identities). Notably, M protein of PPCoV was genetically close to that of RtMruf-CoV-1/JL2014 (87.6% aa identity). However, PPCoV shared the highest (70.7%) aa identity to Alpha-CoV UKRn3 and LRNV Lucheng-19 in N protein (Table 2). NS9 locating downstream from the *N* gene was also found; this is present in some genomes of alphacoronaviruses, such as LRNVs, transmissible gastroenteritis virus (TGEV), porcine respiratory coronavirus (PRCV), and Rhinolophus bat coronavirus HKU2.

### Phylogenetic Relationships

**Figure 2:**
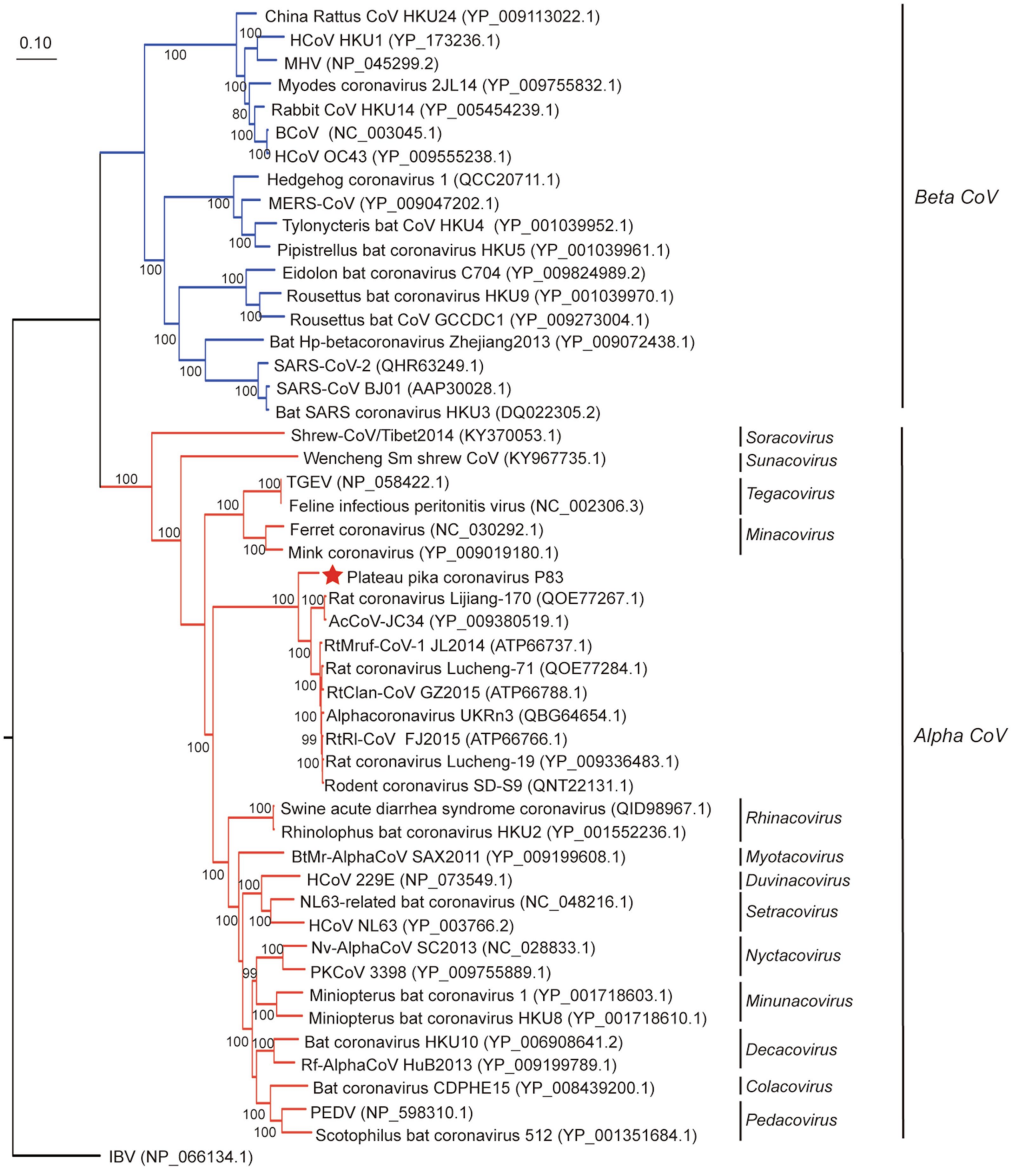
Phylogenetic analysis based on amino acid sequences of ORF1ab. The tree was built by maximum-likelihood method with Dayhoff model and bootstrap values calculated from 1,000 trees. Only bootstrap values >80% are shown. Virus from this study is labeled with a red star. HCoV, human coronavirus; MHV, mouse hepatitis virus; BCoV, bovine coronavirus; MERS-CoV, MERS coronavirus; SARS-CoV, SARS coronavirus; TGEV, porcine transmissible gastroenteritis virus; RtMruf-CoV-1/JL2014, Rodent coronavirus isolate RtMruf-CoV-1/JL2014; RtRl-CoV/FJ2015, Rodent coronavirus isolate RtRl-CoV/FJ2015; AcCoV-JC34, Coronavirus AcCoV-JC34; PKCoV, Alphacoronavirus Bat-CoV/P.kuhlii/Italy/3398; PEDV, Porcine epidemic diarrhea virus.

**Figure 3:**
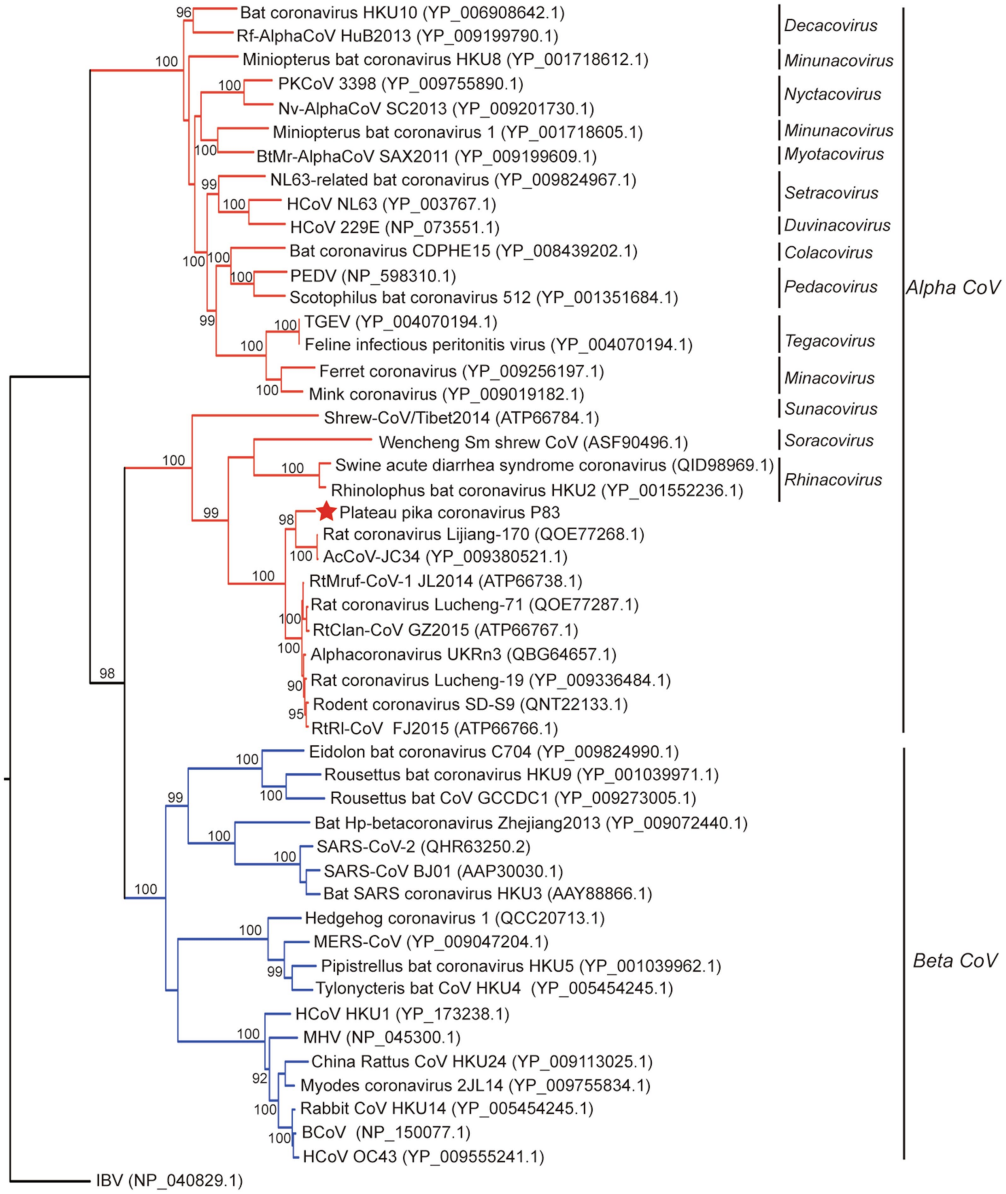
Phylogenetic analysis based on amino acid sequences of the spike protein. The tree was built by maximum-likelihood method with Dayhoff model and bootstrap values calculated from 1,000 trees. Only bootstrap values >90% are shown. The virus from this study is labeled with a red star. HCoV, human coronavirus; MHV, mouse hepatitis virus; BCoV, bovine coronavirus; MERS-CoV, MERS coronavirus; SARS-CoV, SARS coronavirus; TGEV, porcine transmissible gastroenteritis virus; RtMruf-CoV-1/JL2014, Rodent coronavirus isolate RtMruf-CoV-1/JL2014; Rodent coronavirus isolate RtRl-CoV/FJ2015; AcCoV-JC34, Coronavirus AcCoV-JC34; PKCoV, Alphacoronavirus Bat-CoV/P.kuhlii/Italy/3398; PEDV, Porcine epidemic diarrhea virus.

In order to evaluate the evolutionary status of PPCoV, we constructed phylogenetic trees based on aa sequences of ORF1ab, S, M, and N from genera *Alphacoronavirus* and *Betacoronavirus* (Figures 2, 3 and Supplementary Figure S3). The phylogenetic tree of ORF1ab (Figure 2) showed that PPCoV formed a distinct lineage within the genus *Alphacoronavirus*, and was closely related to the cluster of rodent alphacoronaviruses (viruses obtained from rat species; Tsoleridis et al., 2019). A slightly different clustering pattern was observed in phylogenetic trees based on M and N protein sequences, showing that PPCoV was clustered within the rodent coronavirus clade (Supplementary Figure S4).

Evolutionary tree based on S protein sequences (Figure 3) revealed significantly different clustering patterns to phylogenetic trees based on ORF1ab, M, and N proteins. The tree of S indicated that PPCoV formed a divergent cluster with Shrew-CoV/Tibet2014, Wencheng Sm shrew CoV, SADS-CoV, bat coronavirus HKU2, and rodent alphacoronaviruses. However, the clade of the PPCoV as well as other phylogenetically related sub-genera all clustered together with the betacoronaviruses with high bootstrap values.

### Recombination Analysis and Estimation of Divergence Times

All alpha CoVs clustered together in tree of ORF1ab, but some of alpha CoVs clustered together with the beta CoVs in tree of S. This discrepancy in clades between trees suggested a potential recombination for S gene. However, recombination analysis based on S genes of whole set of alpha- and betacoronaviruses indicated that no recombination event was detected for PPCoV (Supplementary Figure S5).

**Figure 4:**
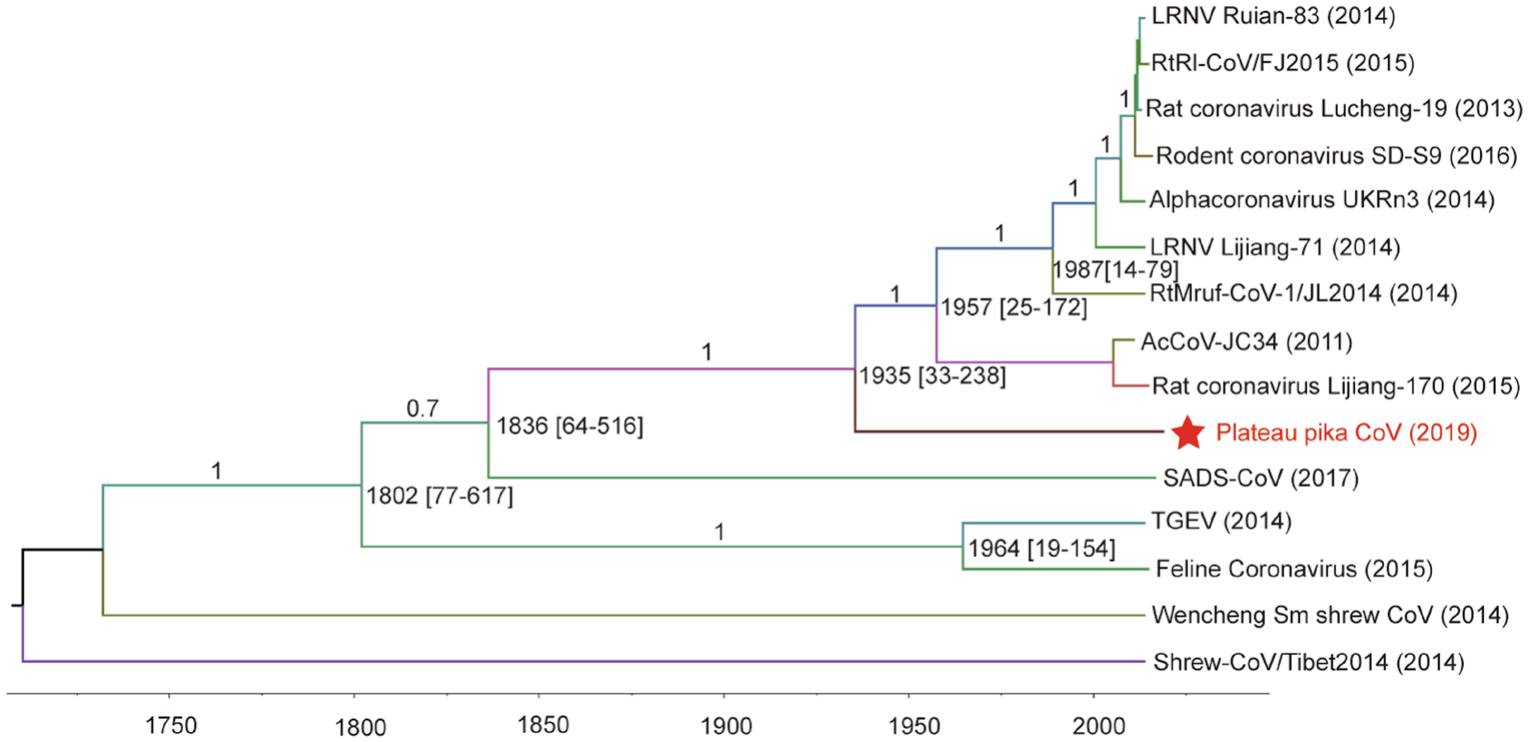
Estimation of the tMRCA of PPCoV based on RdRp genes. The analysis was conducted using BEAST version 1.10 under the SRD06 model and relaxed molecular clock. The mean times of tMRCA are labeled at each node including HPDs in square brackets. The posterior values are labeled at corresponding branches. Brackets after taxa are labeled with sampling time. Labeled nodes are the corresponding mean estimated dates. The virus from this study is labeled with red font and a red star.

For tip-dating analysis, the RdRp and M genes of PPCoV and related alphacoronaviruses were selected and checked for potential recombination regions. Then, the entire sequence alignment (recombination region free) was used to evaluate the divergence time with relaxed clock model as previously described (Lau et al., 2012a,b). The most recent common ancestor (tMRCA) of PPCoV and rodent alphacoronaviruses was estimated to be in the year of 1935 (HPDs 1782 to 1987; approximately 84years ago; Figure 4). The molecular clock analysis based on the M genes also showed the tMRCA of PPCoV and rodent alphacoronaviruses was at 1935 (HPDs 1,420 to 2,008; Supplementary Figure S6). The mean substitution rate of RdRp and M genes was calculated to be 2.1×10^−4^ and 4.8×10^−4^ per site per year, respectively, which is similar to previous report (Woo et al., 2012).

### Virus-Host Co-divergence Analysis

**Figure 5:**
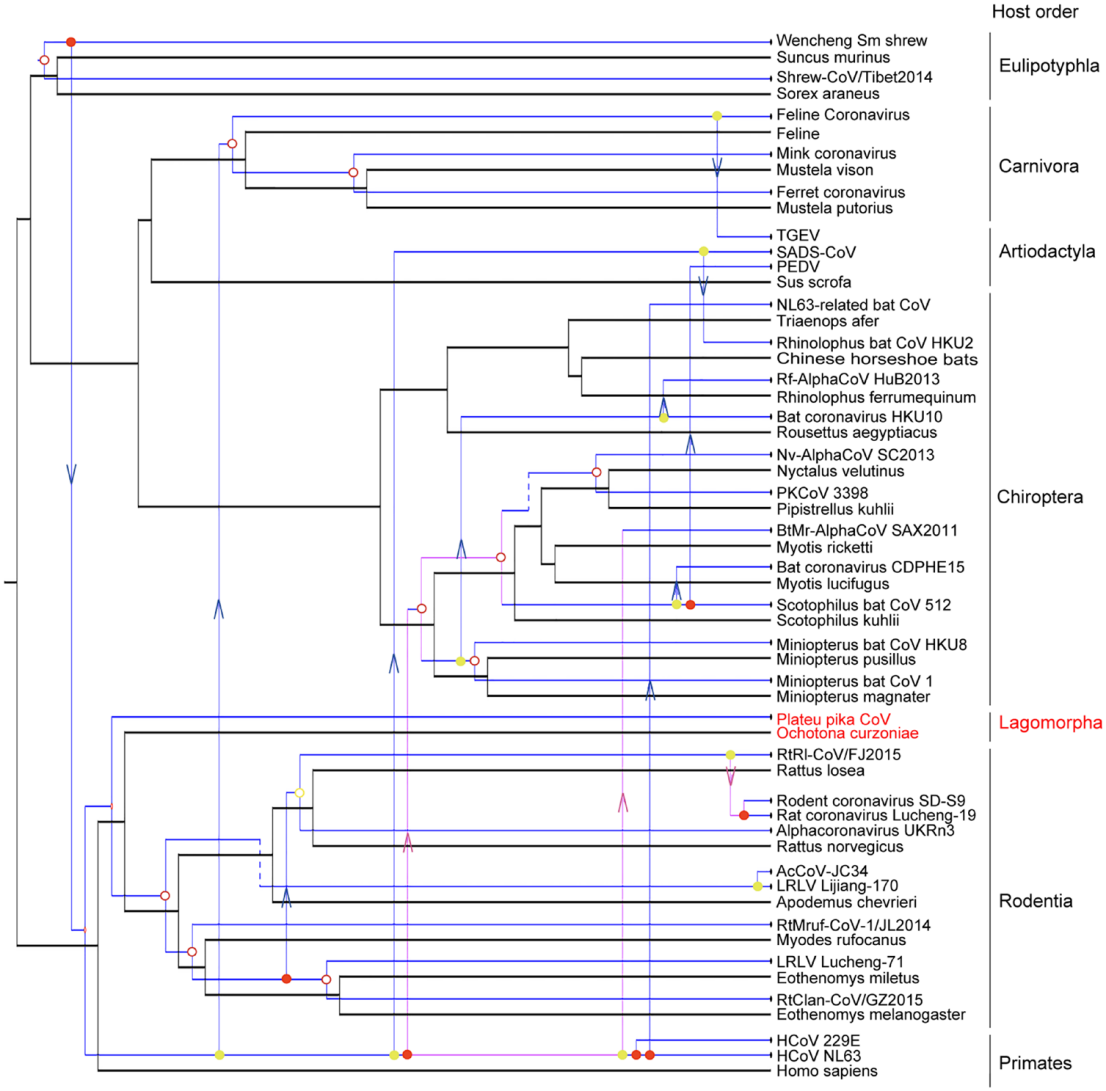
Co-phylogenetic analyses of alphacoronaviruses and their hosts. The host tree is labeled in black with coronavirus tree in blue. Co-divergence events, duplication events, host-switching events, and loss events were labeled with filled circles, empty circles, arrows, and dotted lines, respectively. The virus from this study is labeled with a red font.

The evolutionary history of a virus can mirror that of its host over long-term evolutionary timescales (Shi et al., 2016). A tanglegram was obtained by comparing the tree topologies of viruses and their hosts, which revealed a significant codivergence for alphacoronaviruses (*p*<0.01). The viruses clearly clustered with their hosts at the order level (Figure 5). However, the topology of hosts did not fully match those of viruses with 14 host switching events, two loss events, and three duplication events. Until now, the host species of alphacoronaviruses were clustered into six orders, including orders Artiodactyla, Carnivora, Chiroptera, Eulipotyphla, Rodentia, and Primates (Figure 5). PPCoV was identified in *Ochotona curzoniae* belonging to order Lagomorpha, which is different from host taxon of other alphacoronaviruses. This extends the host range of alphacoronaviruses. Meanwhile, virus-host co-speciation analysis showed that plateau pikas appear to be infected with the ancestor of PPCoV through cross-species transmission (Figure 5), and indicated that the common ancestor for PPCoV and rodent alphacoronaviruses can now trace back to 11.6 (range 0.2–44.6) million years ago.

## Discussion

In this study, a novel alphacoronavirus (PPCoV) was detected and characterized from a new host (plateau pikas) in the Qinghai-Tibet Plateau of China. PPCoV was divergent from known alphacoronaviruses, showing 90% aa identities in three conserved replicase domains (3CLpro, ExoU, and NendoU), and <73.0% aa identities in S protein to other members of genus *Alphacoronavirus*. Moreover, phylogenetic analyses showed that PPCoV clusters together within the clade of rodent CoVs, but separates from the other viruses.

The genome of PPCoV contains an *NS2a* gene encoding a protein homologous to the cyclic phosphodiesterase superfamily. This gene seems to be nonessential for viral replication, but plays a role in pathogenicity of mouse hepatitis virus (MHV; de Haan et al., 2002; Lau et al., 2012b). Further studies are needed to understand the potential function of the NS2a protein. Recently, it was shown that blocking N-glycan biosynthesis will enhance S protein proteolysis, which could decrease SARS-CoV-2 binding to host ACE2 and reduce viral entry (Yang et al., 2020). Thus, the N-glycosylated modifications in S protein of PPCoV may have an important role in viral virulence. By comparing all the trees, and observing discrepancies in clades between trees and values in Table 2, there may be recombination because all alpha CoVs cluster together in tree of ORF1ab, but some of alpha CoVs cluster together with the beta CoVs in tree of S. The identical PPCoV in seven samples indicated that PPCoV is circulating in this site, with a relatively high detection rate of 13.7% in the positive sampling site (Lau et al., 2012b; Wang et al., 2017; Mendenhall et al., 2019). The fact that the respiratory tract of plateau pikas infected with PPCoV showed no obvious pathological change, and that PPCoV was mainly detected in fecal samples with only two positive respiratory samples, suggests a benign enteric tropism for PPCoV, which is similar to that of bat coronavirus HKU10 (Lau et al., 2012a).

Molecular-clock analysis estimates that the tMRCA of PPCoV and rodent alphacoronaviruses emerged around the year of 1935. The virus-host co-speciation analysis shows that alphacoronaviruses co-diverged with their hosts over long-time scales and had a complex evolutionary history with host-switching. Moreover, the study reveals that (1) plateau pikas may have been infected with the common ancestor of PPCoV through cross-species transmission from other unknown mammalian hosts; and (2) the genome of PPCoV represents the first genome of alphacoronaviruses from hosts belonging to the order Lagomorpha, thus broadening the host range of alphacoronaviruses into the seventh order. However, we must acknowledge that the time scales estimated by molecular clock and virus-host co-divergence analyses had a huge discrepancy. There are two possibilities for this discrepancy: (1) the molecular clock is not constant (Holmes, 2003; Sharp and Simmonds, 2011). Because the relative rates of substitution are different between sites along the sequence, and could change dramatically both between viruses and lineages, which lead to a substantial underestimation of divergence times (Holmes, 2003). Also, the genome of PPCoV may have changed by combination of multiple substitution and mutation to adapt to host and/or environmental factors in the long evolutionary history, which influenced the result of tip-dating analysis. Thus, the co-speciation over millions of years between pikas and PPCoV is true. (2) The PPCoV is young as depicted by molecular clocks. It is well-known that cross-species transmissions between closely related host species occurs more easier than between distantly related hosts (Charleston and Robertson, 2002). Thus, PPCoV may in fact infect the pikas through occasional cross-species jump from a closely related animal species recently, and the match between PPCoV and host phylogenies gives a false impression of co-speciation. In addition, a previous report showed that the genus *Alphacoronavirus* may have originated early in Asian house shrews (*Suncus murinus*; Wang et al., 2017). Recently, another phylogenetically distinct genome of alphacoronavirus (Shrew-CoV/Tibet2014) was also identified in shrews that belong to order Eulipotyphla (Wu et al., 2018). Results of tMRCA and co-divergence analyses support shrews as emerging hosts of alphacoronaviruses and suggest that alphacoronaviruses may have emerged from *Sorex araneus*. The role of shrews in alphacoronavirus evolution needs continuous studies.

Although no evidence for recombination was detected for S gene of PPCoV, the recombination of coronaviruses at this site deserves future attention. Previously, we have identified a new deltacoronavirus in birds and marmots at the same sampling site (Zhu et al., 2021b). Interestingly, birds and plateau pikas may inhabit the same caves in the Qinghai-Tibet Plateau of China. Coinfection of different CoVs in the same sites may potentially create opportunities for new recombination and emergence of new viruses.

In conclusion, based on its host, phylogenetic status, and sampling location, PPCoV represents a novel member of the genus *Alphacoronavirus*. This study indicates that plateau pikas may play a significant and previously unrecognized role in the ecology of alphacoronaviruses, and that their role in coronavirus evolution merits further study.

## Data Availability Statement

The viral genome sequences obtained in this study have been deposited in GenBank under accession number MZ577265.

## Ethics Statement

The animal study was reviewed and approved by the Ethics Committee of the National Institute for Communicable Disease Control and Prevention of China CDC.

## Author Contributions

JX contributed to conception and design of the study. WZ, SL, and SW collected the samples. WZ performed the experiments. WZ, JP, ZL, DJ, and X-lL performed the statistical analysis. JX, WZ, and JY wrote the first draft of the manuscript. JX, JY, and LL acquired the funding. All authors contributed to the article and approved the submitted version.

## Funding

This work was supported by grants from National Key R&D Program of China (2019YFC1200501 and 2019YFC1200505) and Research Units of Discovery of Unknown Bacteria and Function (2018RU010).

## Conflict of Interest

The authors declare that the research was conducted in the absence of any commercial or financial relationships that could be construed as a potential conflict of interest.

## Publisher’s Note

All claims expressed in this article are solely those of the authors and do not necessarily represent those of their affiliated organizations, or those of the publisher, the editors and the reviewers. Any product that may be evaluated in this article, or claim that may be made by its manufacturer, is not guaranteed or endorsed by the publisher.

## Footnotes

^1^ https://services.healthtech.dtu.dk/

## References

[ref1] Bankevich A. Nurk S. Antipov D. Gurevich A. A. Dvorkin M. Kulikov A. S.. (2012). SPAdes: a new genome assembly algorithm and its applications to single-cell sequencing. J. Comput. Biol. 19, 455–477. doi: 10.1089/cmb.2012.0021, PMID: 22506599 PMC3342519

[ref2] Blum M. Chang H. Y. Chuguransky S. Grego T. Kandasaamy S. Mitchell A.. (2021). The InterPro protein families and domains database: 20 years on. Nucleic Acids Res. 49, D344–d354. doi: 10.1093/nar/gkaa977, PMID: 33156333 PMC7778928

[ref3] Buchfink B. Xie C. Huson D. H. (2015). Fast and sensitive protein alignment using DIAMOND. Nat. Methods 12, 59–60. doi: 10.1038/nmeth.3176, PMID: 25402007

[ref4] Charleston M. A. Robertson D. L. (2002). Preferential host switching by primate lentiviruses can account for phylogenetic similarity with the primate phylogeny. Syst. Biol. 51, 528–535. doi: 10.1080/10635150290069940, PMID: 12079649

[ref5] Cheng S. Wu H. Chen Z. (2020). Evolution of transmissible gastroenteritis virus (TGEV): a codon usage perspective. Int. J. Mol. Sci. 21:7898. doi: 10.3390/ijms21217898, PMID: 33114322 PMC7660598

[ref6] Conow C. Fielder D. Ovadia Y. Libeskind-Hadas R. (2010). Jane: a new tool for the cophylogeny reconstruction problem. Algorithms Mol. Biol. 5:16. doi: 10.1186/1748-7188-5-16, PMID: 20181081 PMC2830923

[ref7] de Haan C. A. Masters P. S. Shen X. Weiss S. Rottier P. J. (2002). The group-specific murine coronavirus genes are not essential, but their deletion, by reverse genetics, is attenuating in the natural host. Virology 296, 177–189. doi: 10.1006/viro.2002.1412, PMID: 12036329 PMC7133727

[ref8] Gao F. Ou H. Y. Chen L. L. Zheng W. X. Zhang C. T. (2003). Prediction of proteinase cleavage sites in polyproteins of coronaviruses and its applications in analyzing SARS-CoV genomes. FEBS Lett. 553, 451–456. doi: 10.1016/S0014-5793(03)01091-3, PMID: 14572668 PMC7232748

[ref9] Grabherr M. G. Haas B. J. Yassour M. Levin J. Z. Thompson D. A. Amit I.. (2011). Full-length transcriptome assembly from RNA-Seq data without a reference genome. Nat. Biotechnol. 29, 644–652. doi: 10.1038/nbt.1883, PMID: 21572440 PMC3571712

[ref10] Guindon S. Dufayard J. F. Lefort V. Anisimova M. Hordijk W. Gascuel O. (2010). New algorithms and methods to estimate maximum-likelihood phylogenies: assessing the performance of PhyML 3.0. Syst. Biol. 59, 307–321. doi: 10.1093/sysbio/syq010, PMID: 20525638

[ref11] Haake C. Cook S. Pusterla N. Murphy B. (2020). Coronavirus infections in companion animals: virology, epidemiology, clinical and pathologic features. Viruses 12:1023. doi: 10.3390/v12091023, PMID: 32933150 PMC7551689

[ref12] Holmes E. C. (2003). Molecular clocks and the puzzle of RNA virus origins. J. Virol. 77, 3893–3897. doi: 10.1128/JVI.77.7.3893-3897.2003, PMID: 12634349 PMC150674

[ref13] Jiang S. Shi Z. L. (2020). The first disease X is caused by a highly transmissible acute respiratory syndrome coronavirus. Virol. Sin. 35, 263–265. doi: 10.1007/s12250-020-00206-5, PMID: 32060789 PMC7091198

[ref14] Jung K. Saif L. J. Wang Q. (2020). Porcine epidemic diarrhea virus (PEDV): an update on etiology, transmission, pathogenesis, and prevention and control. Virus Res. 286:198045. doi: 10.1016/j.virusres.2020.198045, PMID: 32502552 PMC7266596

[ref15] Kalyaanamoorthy S. Minh B. Q. Wong T. K. F. von Haeseler A. Jermiin L. S. (2017). ModelFinder: fast model selection for accurate phylogenetic estimates. Nat. Methods 14, 587–589. doi: 10.1038/nmeth.4285, PMID: 28481363 PMC5453245

[ref16] Katoh K. Standley D. M. (2013). MAFFT multiple sequence alignment software version 7: improvements in performance and usability. Mol. Biol. Evol. 30, 772–780. doi: 10.1093/molbev/mst010, PMID: 23329690 PMC3603318

[ref17] Krogh A. Larsson B. von Heijne G. Sonnhammer E. L. (2001). Predicting transmembrane protein topology with a hidden Markov model: application to complete genomes. J. Mol. Biol. 305, 567–580. doi: 10.1006/jmbi.2000.4315, PMID: 11152613

[ref18] Kumar S. Stecher G. Suleski M. Hedges S. B. (2017). TimeTree: a resource for timelines, timetrees, and divergence times. Mol. Biol. Evol. 34, 1812–1819. doi: 10.1093/molbev/msx116, PMID: 28387841

[ref19] Langmead B. Salzberg S. L. (2012). Fast gapped-read alignment with bowtie 2. Nat. Methods 9, 357–359. doi: 10.1038/nmeth.1923, PMID: 22388286 PMC3322381

[ref20] Lau S. K. Li K. S. Tsang A. K. Shek C. T. Wang M. Choi G. K.. (2012a). Recent transmission of a novel alphacoronavirus, bat coronavirus HKU10, from Leschenault's rousettes to Pomona leaf-nosed bats: first evidence of interspecies transmission of coronavirus between bats of different suborders. J. Virol. 86, 11906–11918. doi: 10.1128/jvi.01305-12, PMID: 22933277 PMC3486284

[ref21] Lau S. K. Woo P. C. Yip C. C. Fan R. Y. Huang Y. Wang M.. (2012b). Isolation and characterization of a novel Betacoronavirus subgroup A coronavirus, rabbit coronavirus HKU14, from domestic rabbits. J. Virol. 86, 5481–5496. doi: 10.1128/jvi.06927-11, PMID: 22398294 PMC3347282

[ref22] Liu J. Li K. Cheng L. Shao J. Yang S. Zhang W.. (2021). A high-throughput drug screening strategy against coronaviruses. Int. J. Infect. Dis. 103, 300–304. doi: 10.1016/j.ijid.2020.12.033, PMID: 33333250 PMC7832824

[ref23] Martin D. Rybicki E. (2000). RDP: detection of recombination amongst aligned sequences. Bioinformatics 16, 562–563. doi: 10.1093/bioinformatics/16.6.562, PMID: 10980155

[ref24] Mendenhall I. H. Kerimbayev A. A. Strochkov V. M. Sultankulova K. T. Kopeyev S. K. Su Y. C. F.. (2019). Discovery and characterization of novel bat coronavirus lineages from Kazakhstan. Viruses 11:356. doi: 10.3390/v11040356, PMID: 30999711 PMC6521082

[ref25] Potter S. C. Luciani A. Eddy S. R. Park Y. Lopez R. Finn R. D. (2018). HMMER web server: 2018 update. Nucleic Acids Res. 46, W200–w204. doi: 10.1093/nar/gky448, PMID: 29905871 PMC6030962

[ref26] Schoeman D. Fielding B. C. (2019). Coronavirus envelope protein: current knowledge. Virol. J. 16:69. doi: 10.1186/s12985-019-1182-0, PMID: 31133031 PMC6537279

[ref27] Sharp P. M. Simmonds P. (2011). Evaluating the evidence for virus/host co-evolution. Curr. Opin. Virol. 1, 436–441. doi: 10.1016/j.coviro.2011.10.018, PMID: 22440848

[ref28] Shi M. Lin X. D. Tian J. H. Chen L. J. Chen X. Li C. X.. (2016). Redefining the invertebrate RNA virosphere. Nature 540, 539–543. doi: 10.1038/nature20167, PMID: 27880757

[ref29] Su S. Xing G. Wang J. Li Z. Gu J. Yan L.. (2016). Characterization of H7N2 avian influenza virus in wild birds and Pikas in Qinghai-Tibet plateau area. Sci. Rep. 6:30974. doi: 10.1038/srep30974, PMID: 27553660 PMC4995509

[ref30] Suchard M. A. Weiss R. E. Sinsheimer J. S. (2001). Bayesian selection of continuous-time Markov chain evolutionary models. Mol. Biol. Evol. 18, 1001–1013. doi: 10.1093/oxfordjournals.molbev.a003872, PMID: 11371589

[ref31] Talavera G. Castresana J. (2007). Improvement of phylogenies after removing divergent and ambiguously aligned blocks from protein sequence alignments. Syst. Biol. 56, 564–577. doi: 10.1080/10635150701472164, PMID: 17654362

[ref32] Tsoleridis T. Chappell J. G. Onianwa O. Marston D. A. Fooks A. R. Monchatre-Leroy E.. (2019). Shared common ancestry of rodent *Alphacoronaviruses* sampled globally. Viruses 11:125. doi: 10.3390/v11020125, PMID: 30704076 PMC6409636

[ref33] van der Hoek L. Pyrc K. Berkhout B. (2006). Human coronavirus NL63, a new respiratory virus. FEMS Microbiol. Rev. 30, 760–773. doi: 10.1111/j.1574-6976.2006.00032.x, PMID: 16911043 PMC7109777

[ref34] Wang W. Lin X. D. Liao Y. Guan X. Q. Guo W. P. Xing J. G.. (2017). Discovery of a highly divergent coronavirus in the Asian house shrew from China illuminates the origin of the *Alphacoronaviruses*. J. Virol. 91, e00764–e00717. doi: 10.1128/jvi.00764-17, PMID: 28637760 PMC5553162

[ref35] Wang W. Lin X. D. Zhang H. L. Wang M. R. Guan X. Q. Holmes E. C.. (2020). Extensive genetic diversity and host range of rodent-borne coronaviruses. Virus Evol. 6:veaa078. doi: 10.1093/ve/veaa078, PMID: 33318860 PMC7665783

[ref36] Wang Q. Vlasova A. N. Kenney S. P. Saif L. J. (2019). Emerging and re-emerging coronaviruses in pigs. Curr. Opin. Virol. 34, 39–49. doi: 10.1016/j.coviro.2018.12.001, PMID: 30654269 PMC7102852

[ref37] Woo P. C. Lau S. K. Chu C. M. Chan K. H. Tsoi H. W. Huang Y.. (2005). Characterization and complete genome sequence of a novel coronavirus, coronavirus HKU1, from patients with pneumonia. J. Virol. 79, 884–895. doi: 10.1128/JVI.79.2.884-895.2005, PMID: 15613317 PMC538593

[ref38] Woo P. C. Lau S. K. Lam C. S. Lau C. C. Tsang A. K. Lau J. H.. (2012). Discovery of seven novel Mammalian and avian coronaviruses in the genus deltacoronavirus supports bat coronaviruses as the gene source of alphacoronavirus and betacoronavirus and avian coronaviruses as the gene source of gammacoronavirus and deltacoronavirus. J. Virol. 86, 3995–4008. doi: 10.1128/jvi.06540-11, PMID: 22278237 PMC3302495

[ref39] Wu Z. Lu L. Du J. Yang L. Ren X. Liu B.. (2018). Comparative analysis of rodent and small mammal viromes to better understand the wildlife origin of emerging infectious diseases. Microbiome 6:178. doi: 10.1186/s40168-018-0554-9, PMID: 30285857 PMC6171170

[ref40] Wu Y. N. Ma Y. J. Liu W. L. Zhang W. Z. (2019). Modeling the spatial Distribution of Plateau Pika (Ochotona curzoniae) in the Qinghai Lake Basin, China. Animals 9:843. doi: 10.3390/ani9100843, PMID: 31640221 PMC6827031

[ref41] Yang J. Bromage T. G. Zhao Q. Xu B. H. Gao W. L. Tian H. F.. (2011). Functional evolution of leptin of Ochotona curzoniae in adaptive thermogenesis driven by cold environmental stress. PLoS One 6:e19833. doi: 10.1371/journal.pone.0019833, PMID: 21698227 PMC3116822

[ref42] Yang Q. Hughes T. A. Kelkar A. Yu X. Cheng K. Park S.. (2020). Inhibition of SARS-CoV-2 viral entry upon blocking N- and O-glycan elaboration. elife 9:e61552. doi: 10.7554/eLife.61552, PMID: 33103998 PMC7685702

[ref43] Yip C. C. Lam C. S. Luk H. K. Wong E. Y. Lee R. A. So L. Y.. (2016). A six-year descriptive epidemiological study of human coronavirus infections in hospitalized patients in Hong Kong. Virol. Sin. 31, 41–48. doi: 10.1007/s12250-016-3714-8, PMID: 26920709 PMC7090542

[ref44] Yu Z. Cheng K. Sun W. Xin Y. Cai J. Ma R.. (2014). Lowly pathogenic avian influenza (H9N2) infection in plateau pika (Ochotona curzoniae), Qinghai Lake, China. Vet. Microbiol. 173, 132–135. doi: 10.1016/j.vetmic.2014.07.002, PMID: 25069623

[ref45] Zhou P. Fan H. Lan T. Yang X. L. Shi W. F. Zhang W.. (2018). Fatal swine acute diarrhoea syndrome caused by an HKU2-related coronavirus of bat origin. Nature 556, 255–258. doi: 10.1038/s41586-018-0010-9, PMID: 29618817 PMC7094983

[ref46] Zhou Z. J. Qiu Y. Pu Y. Huang X. Ge X. Y. (2020). BioAider: an efficient tool for viral genome analysis and its application in tracing SARS-CoV-2 transmission. Sustain. Cities Soc. 63:102466. doi: 10.1016/j.scs.2020.102466, PMID: 32904401 PMC7455202

[ref47] Zhou J. Sun W. Wang J. Guo J. Yin W. Wu N.. (2009). Characterization of the H5N1 highly pathogenic avian influenza virus derived from wild pikas in China. J. Virol. 83, 8957–8964. doi: 10.1128/JVI.00793-09, PMID: 19553321 PMC2738197

[ref48] Zhu W. Song W. Fan G. Yang J. Lu S. Jin D.. (2021a). Genomic characterization of a new coronavirus from migratory birds in Jiangxi Province of China. Virol. Sin. doi: 10.1007/s12250-021-00402-x, PMID: [Epub ahead of print] 34236588 PMC8264173

[ref49] Zhu W. Yang J. Lu S. Lan R. Jin D. Luo X. L.. (2021b). Beta- and novel delta-coronaviruses are identified from wild animals in the Qinghai-Tibetan Plateau, China. Virol. Sin. 36, 402–411. doi: 10.1007/s12250-020-00325-z, PMID: 33259031 PMC7706178

