# Supplementary material for: Discovery and Evolution of a Divergent Coronavirus in the Plateau Pika From China That Extends the Host Range of Alphacoronaviruses
Source: Front Microbiol. 2021 Oct 7;12:755599. doi: 10.3389/fmicb.2021.755599 (PMC8529330; doi:10.3389/fmicb.2021.755599)
Supplement: Supplementary file 1 [file Data_Sheet_1.docx]

**Discovery and Evolution of a Divergent Coronavirus in the Plateau Pika from China** **that Extends the Host Range of Alphacoronaviruses**

Wentao Zhu^1^, Jing Yang^1,2,3^, Shan Lu^1,2,3^, Dong Jin^1,2,3^, Shusheng Wu^4^, Ji Pu^1^, Xue-lian Luo^1^, Liyun Liu^1^, Zhenjun Li^1^ and Jianguo Xu^1,2,3,5*^

1. State Key Laboratory of Infectious Disease Prevention and Control, National Institute for Communicable Disease Control and Prevention, Chinese Center for Disease Control and Prevention, Changping, Beijing 102206, PR China;

2. Shanghai Public Health Clinical Center, Fudan University, Shanghai 201508, PR China;

3. Research Units of Discovery of Unknown Bacteria and Function, Chinese Academy of Medical Sciences, Beijing 100730, PR China;

4. Yushu Prefecture Center for Disease Control and Prevention, Yushu 815000, China;

5. Research Institute of Public Heath, Nankai University, Tianjin 300071, China

*Correspondence author: Jianguo Xu; Email: xujianguo@icdc.cn

Table S1 Characteristics of the putative nonstructural proteins of ORF1ab of PPCoV

| Gene | Putative function  or domain(s) | First amino  acid residue^b^ | Last amino  acid residue^b^ | Length  (aa) | Length of corresponding protein in Lijiang-170 / AcCoV-JC34 |
| --- | --- | --- | --- | --- | --- |
| nsp1 | Leader protein | M^1^ | G^219^ | 219 | 218 (219 in AcCoV-JC34) |
| nsp2 | PL1pro, PL2pro, AC, ADRP, HD | K^220^ | A^2168^ | 1949 | 2059 (2156 in AcCoV-JC34) |
| nsp3 | HD | N^2169^ | V^2679^ | 511 | 508 (509 in AcCoV-JC34) |
| nsp4 | 3CL^pro^ | N^2680^ | Q^3040^ | 361 | 257 |
| nsp5 | HD | I^3041^ | Q^3316^ | 276 | 277 |
| nsp6 | Unknown | R^3317^ | Q^3434^ | 118 | 118 |
| nsp7 | Unknown | S^3435^ | Q^3712^ | 278 | 277 (278 in AcCoV-JC34) |
| nsp8 | Unknown | N^3713^ | Q^3819^ | 107 | 105 |
| nsp9 | Unknown | A^3820^ | Q^3954^ | 135 | 135 |
| nsp10 | RdRp | S^3955^ | Q^4880^ | 926 | 926 |
| nsp11 | Hel | S^4881^ | Q^5477^ | 597 | 597 |
| nsp12 | ExoN | A^5478^ | Q^5996^ | 519 | 520 |
| nsp13 | ExoN | G^5997^ | Q^6341^ | 345 | 346 |
| nsp14 | 2’-*O*-MT | S^6342^ | K^6642^ | 301 | 302 |

PL1Pro and PL2Pro, papain-like protease 1 and 2, respectively; AC, acidic domain; ADRP, ADP-ribose 1-phosphatase; HD, hydrophobic domain; 3CL^pro^, 3C-like protease; RdRp, RNA-dependent RNA polymerase; Hel, helicase; ExoN, 3’-to-5’ exonuclease; NendoU, nidoviral uridylate-specific endoribonuclease; 2’-O-MT, ribose-2’-O-methyltransferase.

Table S2 The primers were used to confirm and screen in this study.

| Primer names | Primer sequences | Targeted region | Length of amplified region |
| --- | --- | --- | --- |
| Alpha-F1 | GTTAGAGATACTGGCACTGTTGT | *NS2* and *spike* genes | 1967 bp |
| Alpha-R1 | GCAACCTTTGGTAATGGAAT |  |  |
| Alpha-F2 | AAGTGTAATGGTTTGGATGTTT | *spike* gene | 1647 bp |
| Alpha-R2 | AGAACTTTGAGCATAAACACATT |  |  |
| Alpha-F3 | TGTGAAAAGTAAAGCAGGTGAT | *NS7a*, *nucleocapsid* *NS9* genes | 2180 bp |
| Alpha-R3 | TTGGAAACTCACATAGAATAAACAT |  |  |


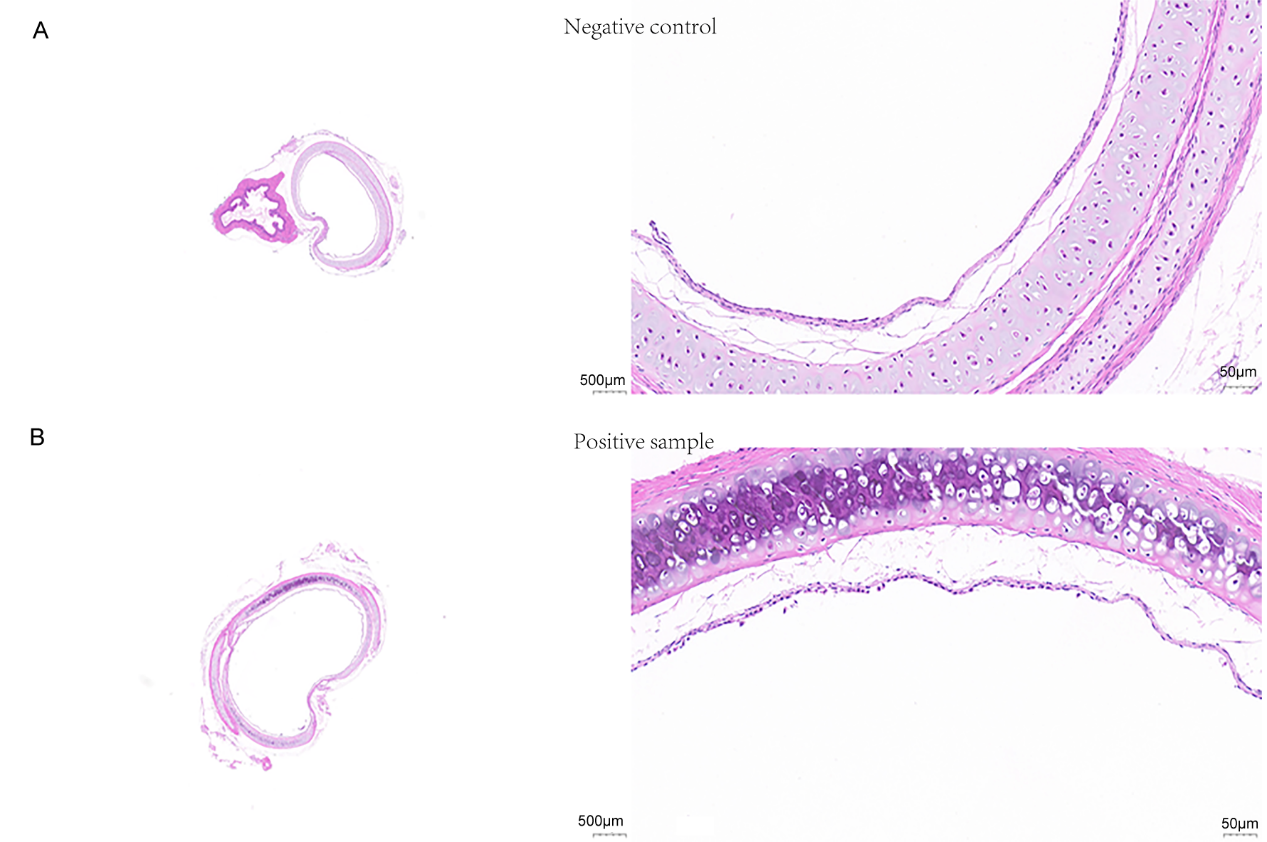


Fig. S1 Histopathology of respiratory tract of plateau pika infected with PPCoV. (A) (A) Mock-infected respiratory tract of plateau pika shows a normal appearance. (B) PPCoV infected respiratory tract with no obvious inflammation. The left picture displays transection of respiratory tract, with right picture zoomed in 10 times. Scale bar is labeled at lower right corner.


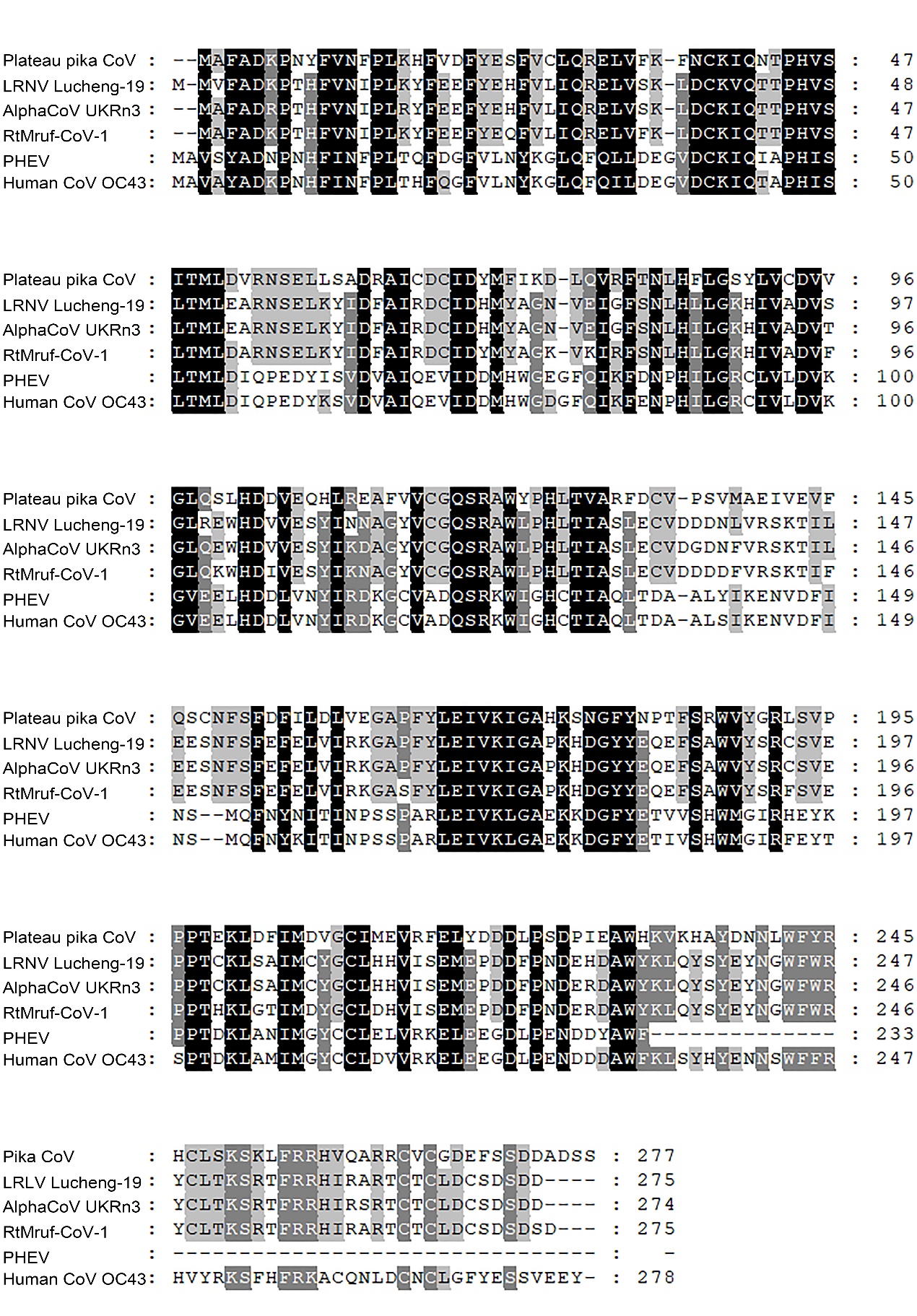


Fig. S2 Multiple alignment of predicted amino acid sequences of NS2a proteins of PPCoV compared to the corresponding NS2a of related coronaviruses. Identical and highly conserved amino acid residues among all viruses are highlighted in black and gray, respectively. Amino acid sites are labeled at right hand side of each row. PHEV, porcine hemagglutinating encephalomyelitis virus. LRNV, Lucheng Rn rat coronavirus.


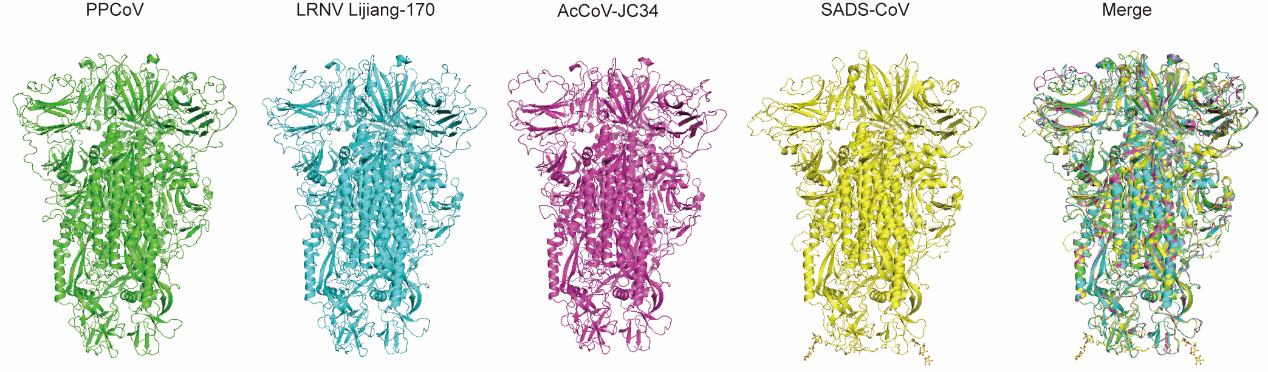


Fig. S3 Homology modelling structures of spike proteins. The three-dimensional structures were modelled using the Swiss-Model platform. PPCoV, plateau pika coronavirus; LRNV, Lucheng Rn rat coronavirus; AcCoV-JC34, Coronavirus AcCoV-JC34; SADS-CoV, Swine acute diarrhea syndrome coronavirus.


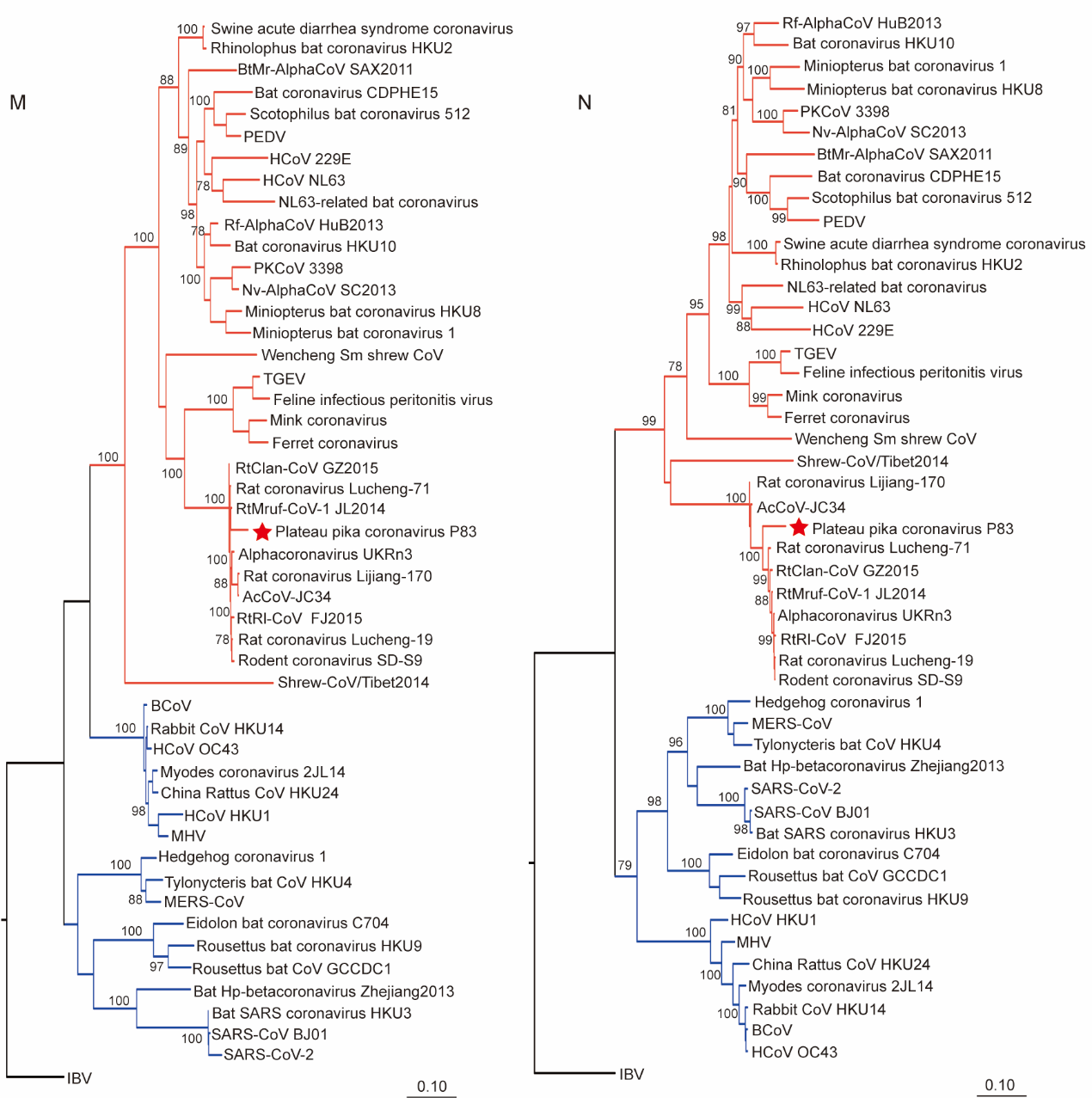


Fig. S4 Phylogenetic analysis based on amino acid sequences of the M and N proteins, respectively. The tree was built by maximum-likelihood method with Dayhoff model and bootstrap values calculated from 1,000 trees. The best substitution model (Dayhoff) was used with 1000 replicates. Numbers on each branches exhibits the percentage that the branch was found in 1000 bootstrap replicates; with only values >70% are shown. The scale bar represents the number of amino acid substitutions per site. The virus from this study is labeled with a red star. HCoV, human coronavirus; MHV, mouse hepatitis virus; BCoV, bovine coronavirus; MERS-CoV, MERS coronavirus; SARS-CoV, SARS coronavirus; TGEV, porcine transmissible gastroenteritis virus; RtMruf-CoV-1/JL2014, Rodent coronavirus isolate RtMruf-CoV-1/JL2014; AcCoV-JC34, Coronavirus AcCoV-JC34; PKCoV, Alphacoronavirus Bat-CoV/P.kuhlii/Italy/3398; PEDV, Porcine epidemic diarrhea virus.


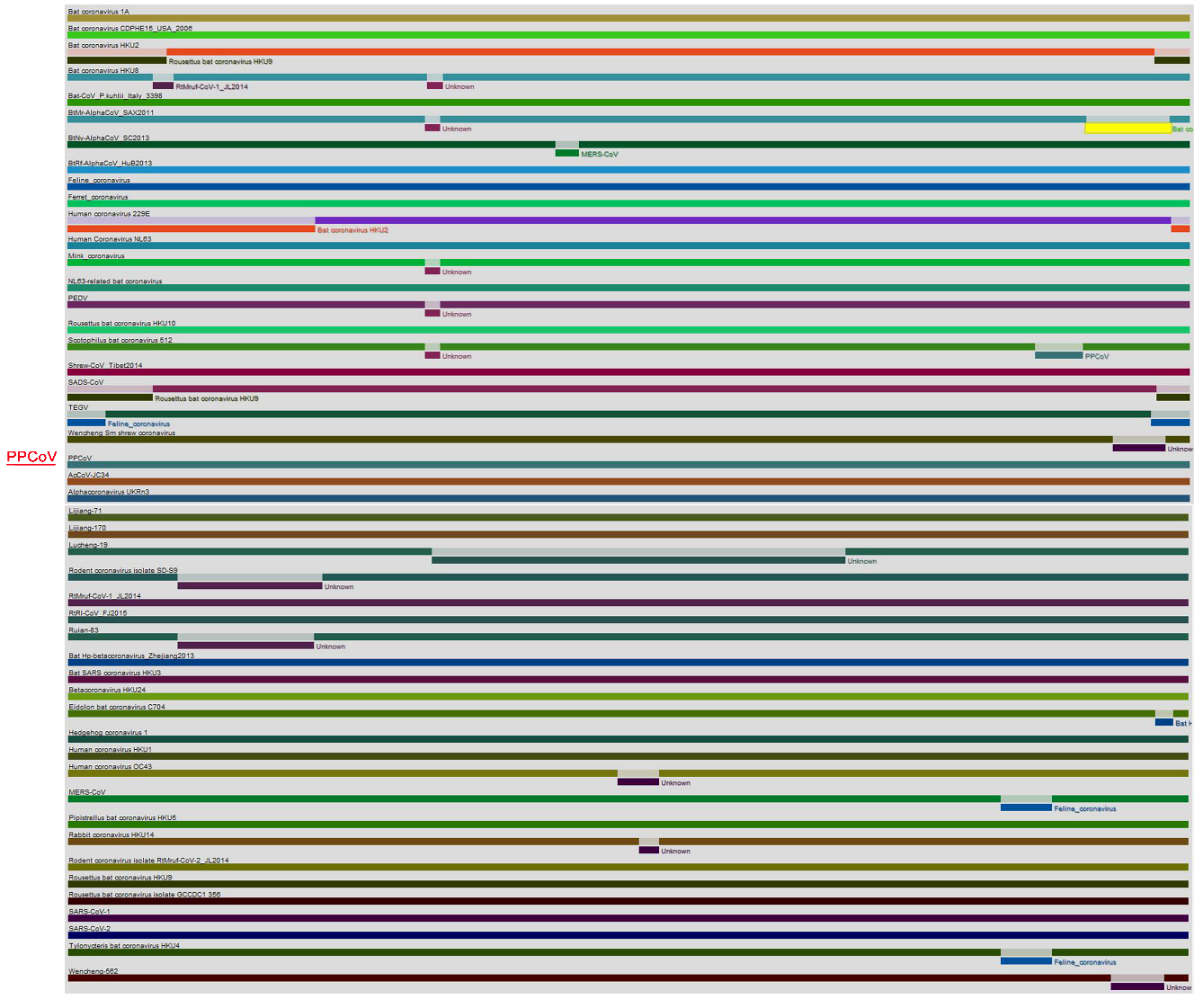


Fig. S5 Detection of potential recombination events of S gene of PPCoV. The recombination analysis was detected by RDP 4 with default parameters. The GenBank accession numbers used were identical with those used in Figure 3. LRNV, Lucheng Rn rat coronavirus; Lijiang-170, LRNV Lijiang-170; Lucheng-19, LRNV Lucheng-19; Lucheng-71, LRNV Lucheng-71; PEDV, porcine epidemic diarrhea virus; SADS-CoV, swine acute diarrhea syndrome coronavirus; HCoV, human coronavirus; MHV, mouse hepatitis virus; BCoV, bovine coronavirus; MERS-CoV, MERS coronavirus; SARS-CoV, SARS coronavirus; AcCoV-JC34, Coronavirus AcCoV-JC34.


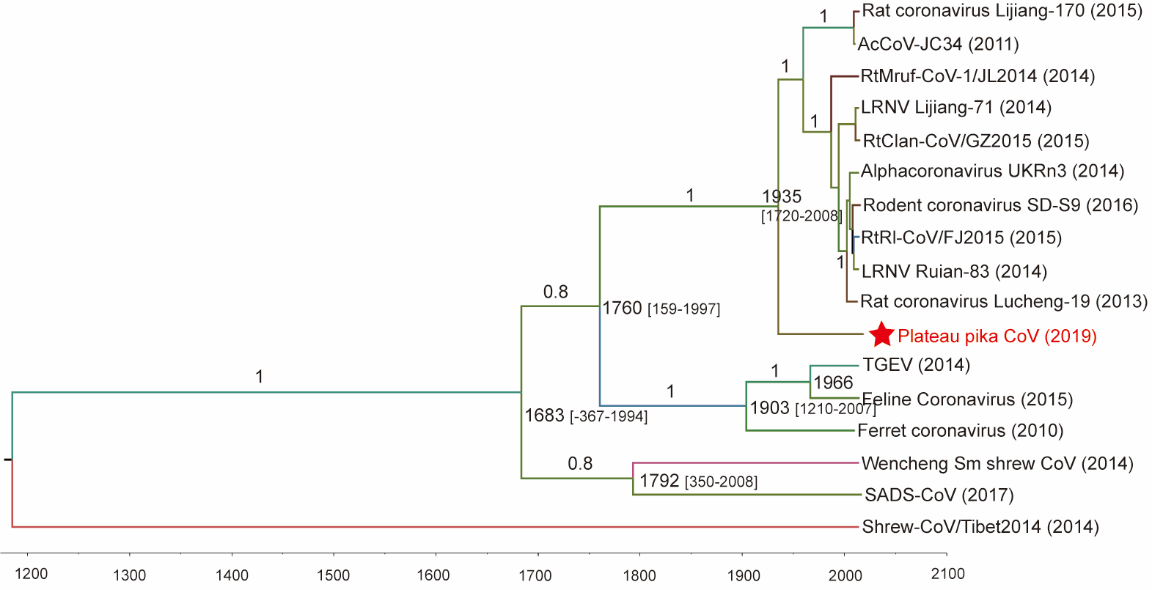


Fig. S6 Estimation of the tMRCA of PPCoV based on membrane genes. The analysis was conducted using BEAST version 1.10 under the relaxed-clock model. The mean times of tMRCA are labeled at each node including HPDs in square brackets. The posterior values are labeled at corresponding branches. Brackets after taxa are labeled with sampling time. Labeled nodes are the corresponding mean estimated dates. The virus from this study is labeled with red font and a red star.
